# Structural Analysis of the Ancestral Haloalkane Dehalogenase AncLinB-DmbA

**DOI:** 10.3390/ijms222111992

**Published:** 2021-11-05

**Authors:** Andrii Mazur, Pavel Grinkevich, Radka Chaloupkova, Petra Havlickova, Barbora Kascakova, Michal Kuty, Jiri Damborsky, Ivana Kuta Smatanova, Tatyana Prudnikova

**Affiliations:** 1 Faculty of Science, University of South Bohemia in Ceske Budejovice, Branisovska 1760, 370 05 Ceske Budejovice, Czech Republic; mazura00@prf.jcu.cz (A.M.); pavel.grinkevich@gmail.com (P.G.); havlip04@prf.jcu.cz (P.H.); karafb00@prf.jcu.cz (B.K.); kutym@seznam.cz (M.K.); 2 Loschmidt Laboratories, Department of Experimental Biology and RECETOX, Faculty of Science, Masaryk University, Kamenice 5, 625 00 Brno, Czech Republic; radka@chemi.muni.cz (R.C.); jiri@chemi.muni.cz (J.D.); 3 Enantis Ltd., Kamenice 771/34, 625 00 Brno, Czech Republic; 4 International Clinical Research Center, St Anne’s University Hospital Brno, Pekarska 53, 656 91 Brno, Czech Republic

**Keywords:** haloalkane dehalogenase, ancestral sequence reconstruction, structural analysis, halogenated pollutants

## Abstract

Haloalkane dehalogenases (EC 3.8.1.5) play an important role in hydrolytic degradation of halogenated compounds, resulting in a halide ion, a proton, and an alcohol. They are used in biocatalysis, bioremediation, and biosensing of environmental pollutants and also for molecular tagging in cell biology. The method of ancestral sequence reconstruction leads to prediction of sequences of ancestral enzymes allowing their experimental characterization. Based on the sequences of modern haloalkane dehalogenases from the subfamily II, the most common ancestor of thoroughly characterized enzymes LinB from *Sphingobium japonicum* UT26 and DmbA from *Mycobacterium bovis* 5033/66 was in silico predicted, recombinantly produced and structurally characterized. The ancestral enzyme AncLinB-DmbA was crystallized using the sitting-drop vapor-diffusion method, yielding rod-like crystals that diffracted X-rays to 1.5 Å resolution. Structural comparison of AncLinB-DmbA with their closely related descendants LinB and DmbA revealed some differences in overall structure and tunnel architecture. Newly prepared AncLinB-DmbA has the highest active site cavity volume and the biggest entrance radius on the main tunnel in comparison to descendant enzymes. Ancestral sequence reconstruction is a powerful technique to study molecular evolution and design robust proteins for enzyme technologies.

## 1. Introduction

Halogenated compounds are major components of herbicides, insecticides, fungicides, and other chemical agents that are widespread in industry and agriculture. Their environmentally safe disposal still remains an unsolved issue to date [1]. Haloalkane dehalogenases (HLDs) (EC 3.8.1.5) are hydrolytic enzymes that cleave carbon-halogen bonds in a broad range of halogenated aliphatic compounds, resulting in a corresponding alcohol, a halide ion, and a proton [2,3,4]. The interest in these enzymes is growing because of their utilization in biocatalysis, bioremediation, biosensing, and molecular imaging [5]. Structurally, HLDs belong to the α/β-hydrolase fold superfamily [6,7]. The tertiary structure of HLDs is comprised of two domains: an α/β-hydrolase core domain and a helical cap domain. The α/β-hydrolase domain is composed of an eight-stranded mostly parallel β-sheet flanked by α-helices and serves as a scaffold for the catalytic residues. The cap domain consists of a few helices inserted in the catalytic domain and is known to influence the substrate specificity of these enzymes [8]. The active site cavity is located between the core main domain and the cap domain [9]. HLDs are divided into three subfamilies, HLD-I, HLD-II, and HLD-III, according to the composition of their catalytic residues and the anatomy of the cap domain. The catalytic residues in the HLD-I subfamily are D-H-D/W-W, while HLD-II members predominantly contain D-H-E/N-W and HLD-III members contain D-H-D/N-W [10,11].

LinB from *Sphingobium japonicum* UT26 [12,13] and DmbA from *Mycobacterium bovis* 5033/66 [14] are closely related enzymes sharing 68% of sequence identity [15] and both exhibiting a wide range of substrate specificity. The LinB breaks down many halogenated substrates including 1,2-dibromoethane, 1,3-dibromopropane, 1-bromo-3-chloropropane, 1-bromo-2-chloroethane, 4-bromobutanenitrile, γ-hexachlorocyclohexane and 1,2-dibromoethane [15,16]. It has been shown that its active site is one of the largest among other HLDs [17]. DmbA has a similar catalytic behavior as LinB but differs in access tunnels composition. It also catalyzes biodegradation of 1,2-dichloroethane, 2-iodobutane and 1,3-dichloropropene [15,18]. These properties make LinB and DmbA a good target for protein modification and bioengineering. A number of these enzyme variants have been successfully crystallized and their crystal structures have been solved [19,20,21,22].

Most of the enzymes have their active sites buried inside the protein core, rather than exposed to the solvent on the surface. These buried active sites are connected to the bulk solvent through tunnels, which act as exchange pathways for the substrate penetration and product release from the active site [23]. The structural and biochemical properties of these tunnels have a major impact on the catalysis and substrate specificity of an enzyme [24]. Therefore, the access tunnels became a frequent target of enzyme modifications [21]. Haloalkane dehalogenases contain several tunnels connecting the protein surface and the active site cavity. The tunnels identified in crystal structures of HLDs are usually referred to as the main tunnel and several slot tunnels; each of them can serve as a path for the substrate access to the active site, release of the reaction products from the active site, and exchange of solvent molecules. The main p1 tunnel is used for the halogenated substrate access to the active site as well as alcohol and halide products exchange. The p2 secondary slot tunnels are served for the alcohol release and water solvent exchange. There are several further variations of tunnel branches originating from p1 and p2 tunnels [21,23,25].

Ancestral sequence reconstruction represents a valuable tool for modification of the structure and biochemical properties of modern enzymes [26]. This technique allows the prediction of the sequence of a hypothetical common ancestor from an already known and related set of sequences of modern-day enzymes. Gene-encoding inferred ancestral sequence can then be synthetized, expressed in expression systems, and characterized. Here we report crystallization and X-ray structural analysis of the ancestral enzyme AncLinB-DmbA that combines structural features of LinB, DmbA and other closely related modern haloalkane dehalogenases.

## 2. Results and Discussion

A freshly isolated and purified sample of AncLinB-DmbA in a concentration of 9.8 mg/mL in 50 mM Tris–HCl buffer pH 7.5 was used for the initial screening of suitable crystallization conditions. The initial crystallization screen resulted in the formation of several needle-shaped and 3D crystals. Three-dimensional (3D) crystals grew in the condition with precipitant composed of 30% PEG 4000, 0.1 M sodium citrate pH 5.6, 0.2 M ammonium acetate. Several crystals were prepared for diffraction analysis on the synchrotron. The diffraction dataset was collected at the resolution of 1.5 Å. The data collection statistics are presented in Table 1. The tetragonal *P*4_3_22 space group was determined in POINTLESS [27] from CCP4 [28] program suit for further data processing. The model was constructed using molecular replacement and the structure of DmbA (PDB ID 2QVB) [14] sharing 83.2% sequence identity with AncLinB-DmbA was used as a template. After several cycles of restrained and anisotropic refinement as well as a manual rebuilding and refinement in COOT [29] the *R_work_* and *R_free_* of 15.9% and 17.1%, respectively, were achieved.

**Figure 1:**
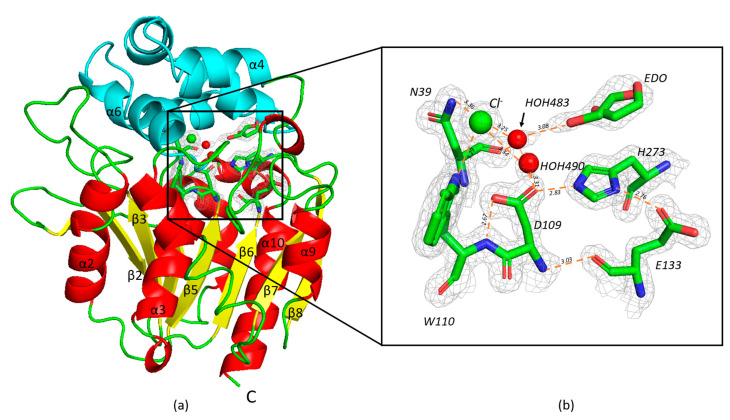
Cartoon model of the overall structure of AncLinB-DmbA (**a**) and the active site with bound ethylene glycol (EDO) (**b**). The overall structure of AncLinB-DmbA (**a**) is shown in cartoon representation. α-helices are shown in red; β-strands are in yellow, and loops are in green for the core domain; the cap domain is shown in cyan. Details of the active site (**b**): the catalytic pentad and EDO are represented by sticks; the chloride ion and water molecules are shown as green and red spheres, respectively; coordinating interactions are represented by orange dashed lines; the *2Fo-Fc* electron density map for ions and interacting residues contoured at 1.3σ is drawn as light grey mesh.

The structural organization of AncLinB-DmbA is similar to other HLDs-II subfamily members [9,10]; it is composed of two compact domains, a cap domain and a main domain. The cap domain is responsible for the substrate specificity, while the main domain carries out the catalytical function (Figure 1a). Eight β-strands, where β2 is antiparallel, form a twisted β-sheet surrounded by six α-helices on both sides. The α2, α3, α8, and α9 are located on the front side and the others on the back side. The cap domain consists of residues 149 to 211. It is formed by α4, α5, α6, and α7 helices and connects to the main domain before α8 (Figure 1a).

The active site of AncLinB-DmbA is similar to LinB, DmbA, and other HLD-II members [10]. It is buried in a hydrophobic cavity between two domains (Figure 1b). The catalytic triad contains nucleophile D109, catalytic acid E133, and catalytic base H273. W110 and N39 act as halide-stabilizing residues in AncLinB-DmbA (Figure 1b). All together three catalytic and two halide-binding amino acids represent the catalytic pentad. The electron density map in the vicinity of the active site was interpreted as a chloride anion. coordinated by two water molecules (HOH483 and HOH490) and the ethylene glycol molecule (EDO) as a component of the precipitant in two alternative conformations (Figure 1b).

The chloride anion is coordinated by two halide stabilizing residues with distances between the Nε1 atom in W110 and the Nδ1 atom in N39 of 3.29 and 3.37 Å, respectively. Further coordination of chloride ion is provided by N atom from P209 with 3.56 Å distance and two water molecules HOH483 and HOH490 with 3.25 and 3.34 Å distance, respectively (Figure 1b). The catalytic pentad is stabilized by the following hydrogen bonds: the N atom of the E109 interacts with the O atom of E133 with 3.03 Å distance, the D109 Oδ1 atom is coordinated by the Nε1 of H273 with a distance of 2.83 Å, and H273 Nδ1 interacts with D109 Oδ1 with Oε1 atom of E133 with 2.83 Å and 2.76 Å distances, respectively.

**Figure 2:**
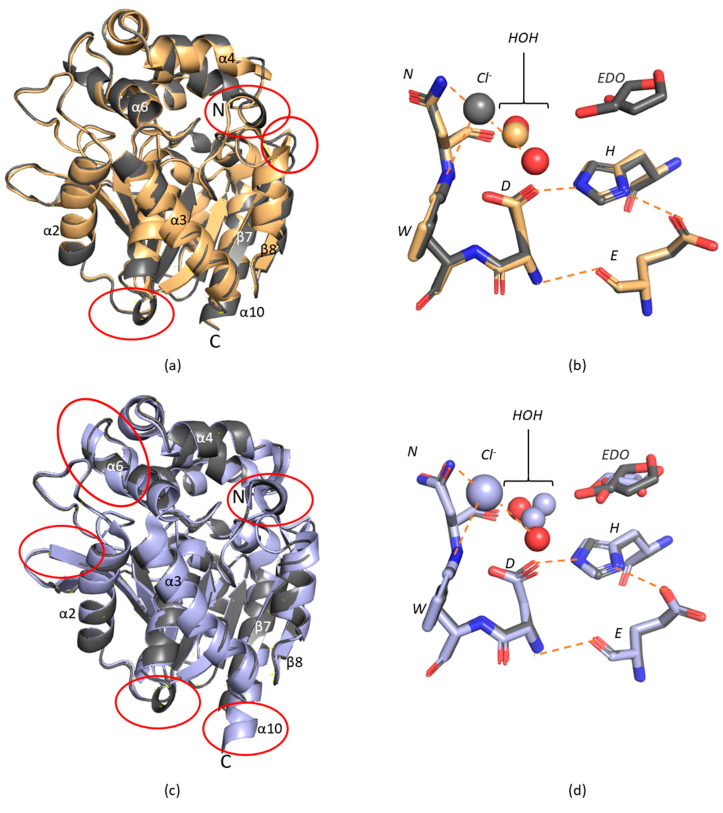
Structural comparison of AncLinB-DmbA (PDB ID 7PW1), LinB (PDB ID 1CV2) and DmbA (PDB ID 2QVB). (**a**,**c**) Superposition of the overall structure of AncLinB-DmbA with LinB (**a**) and of AncLinB-DmbA with DmbA (**c**). Cα cartoon trace shows elements of the protein secondary structures. The AncLinB-DmbA is colored in grey; LinB is colored in light brown; DmbA is shown in light blue. The most significant structural differences are highlighted by red ellipses. (**b**,**d**) Superposition of AncLinB-DmbA with LinB (**b**) and AncLinB-DmbA with DmbA (**d**) active sites. Amino acids of AncLinB-DmbA are shown as grey sticks; amino acids of LinB are shown as light-yellow sticks; amino acids of DmbA are shown as light-blue sticks. The ions and other ligands are presented in the same color for related proteins.

The 3D structure of AncLinB-DmbA was superposed with its closely related structures of descendant enzymes LinB and DmbA. As it is shown in Figure 2a the enzyme structure is highly conserved (RMSD of Cα atoms of AncLinB-DmbA and LinB is 0.778 Å and AncLinB-DmbA and DmbA is 0.895 Å), however, some small differences between the structure of ancestral and descendant enzymes were identified. These differences were uncovered not only in the cap domain but also in the main domain (Figure 2a). The α4 in the cap domain of AncLinB-DmbA is shorter than the corresponding helices of LinB and DmbA and has a slightly different position; moreover, α6 in the cap domain of DmbA is longer than the corresponding helices of AncLinB-DmbA and LinB (Figure 2a). Both these differences are presented in the positions, where the cap domain connects with a core domain. Furthermore, some differences are presented in several loops: the loop near the N-terminus of AncLinB-DmbA forms an additional small helix and another additional helix is formed in AncLinB-DmbA between α3 and β6, which is not presented in DmbA and LinB structures. The core domain structure is generally more conserved among all compared enzymes, however, some structural differences in the arrangement of the secondary elements were also observed. The β1, β2, and β7 are much shorter in AncLinB-DmbA than in DmbA and LinB. The β1 differs in AncLinB-DmbA not only by length but also by an angle that is almost orthogonal to the position of β1 in LinB and DmbA. The β8 sheet in AncLinB-DmbA is shorter than in the other two proteins and slightly shifted by 3 Å towards α10 helix than the corresponding sheet in both modern enzymes. The α10 helix is longer in DmbA by five amino acids compared to other enzymes (Figure 2a).

The active site is located at the same position in all compared proteins (Figure 2b). The catalytic amino acids are conserved. The water molecules have similar distances to the corresponding coordinating residues in all tested enzymes. The chloride ion is presented in all structures at a similar position. Similarly, to AncLinB-DmbA, ethylene glycol in two conformations was found in the active site of DmbA as an artifact of used crystallization conditions. This leads to the similarities in bond distances of water molecules in both DmbA and AncLinB-DmbA.

**Figure 3:**
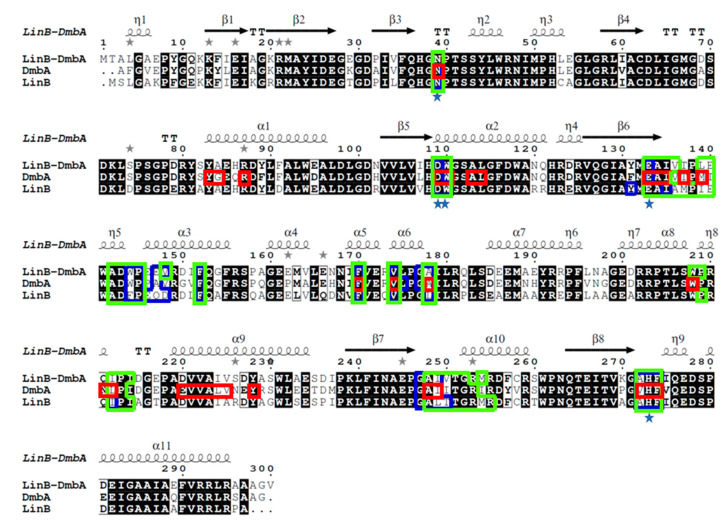
Sequence alignment of ancestor AncLinB-DmbA with modern-day counterparts LinB from *Sphingobium japonicum* UT26 (PDB ID 1CV2) and DmbA from *Mycobacterium bovis* 5033/66 (PDB ID 2QVB). Amino acid residues forming the p1 tunnel are marked in blue box, the residues of the p2a tunnel are marked in green box and the residues of the p2b tunnel are marked in red box. Catalytic residues are marked by blue stars. Identical residues are highlighted by black background; similar residues are shown in bold typeface. The alignment was generated with Clustal Omega [31] and visualized using ESPript 3.0 [32].

The sequence alignment of AncLinB-DmbA (PDB ID 7PW1), LinB from *Sphingobium japonicum* UT26 (PDB ID 1CV2) [12]) and DmbA from *Mycobacterium bovis* 5033/66 (PDB ID 2QVB) [14]) was performed to compare AncLinB-DmbA with related dehalogenases in their access tunnel composition (Figure 3). The alignment shows a highly conserved secondary structure of AncLinB-DmbA to the modern dehalogenases. The active site residues are conserved among all compared proteins; the catalytic triad D109, E133, and H273 (numbers according to AncLinB-DmbA) and two halide-binding residues (N39 and W110) are identical in the tested dehalogenases (Figure 3), which is in agreement with the whole HLD-II subfamily [10,15,30]. However, comparison to the residues involved in access tunnel composition revealed some differences. The main differences in the composition of the access tunnels between the AncLinB-DmbA and the modern enzymes were found in positions 147, 135–139, and 250. The residue corresponding to position 147 in AncLinB-DmbA plays an important functional role, due to its location in the mouth of the p1 tunnel. AncLinB-DmbA contains glutamic acid in position 147, while DmbA contains alanine and LinB contains glutamine in the corresponding position. Another difference in tunnel composition was identified in position 250, where valine is found in AncLinB-DmbA, isoleucine in DmbA, and threonine in LinB. This position is also functionally important due to its location at the entrance of the p2 tunnel.

**Figure 4:**
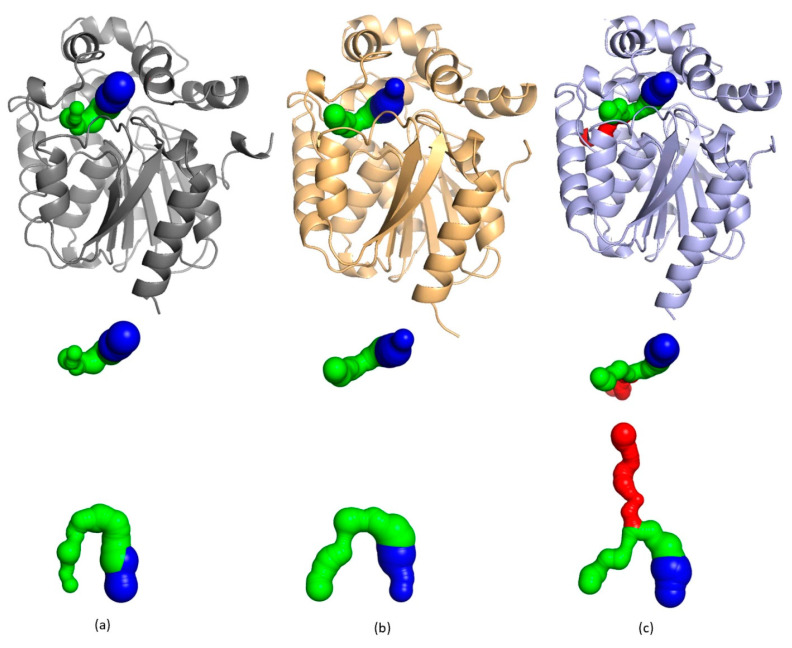
Access tunnels identified in crystal structure of AncLinB-DmbA (PDB ID 7PW1) (**a**), LinB (PDB ID 1CV2) (**b**) and DmbA (PDB ID 2QVB) (**c**). The crystal structures are presented in cartoon representation. Access tunnels are presented in two orientations: front (up) and top (down) view. The main (p1) tunnel is presented in blue, and slot p2a and p2b tunnels are presented in green and red, respectively.

The active site cavity is connected to the solvent on the protein surface by several tunnels. The physical properties of these tunnels play an important role in the substrate specificity of the enzyme [33]. The tunnel architecture was calculated using the program Caver WEB [34], and some differences in the tunnel and cavity constructions were found between the compared proteins (Figure 4). The AncLinB-DmbA and LinB contain one main tunnel p1 (highlighted in blue) and one slot tunnel p2a, while DmbA has one main tunnel p1 and two slot tunnels p2a and p2b (Figure 4). The information about the tunnel composition of the tested enzymes is listed in Table 2.

**Figure 5:**
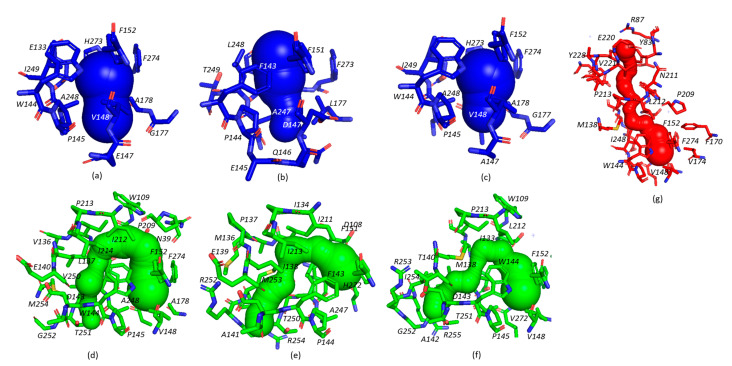
The p1 and p2 access tunnels with corresponding amino acids of AncLinB-DmbA (PDB ID 7PW1) (**a**,**d**), LinB (PDB ID 1CV2) (**b**,**e**) and DmbA (PDB ID 2QVB) (**c**,**f**,**g**). The main p1 tunnel with its residues is colored blue, p2a slot tunnel with amino acids is colored green, p2b tunnel with its amino acids is colored red.

The p1 tunnel of AncLinB-DmbA is formed by 17 residues. The bottleneck consists of residues P145, E147, V148, V174, A178, A272, G177, and its radius is ∼2 Å (Figure 5a). The largest bottleneck radius of p1 is determined to be ∼2.10 Å in DmbA (Figure 5c). The smallest radius is in LinB (∼1.45 Å on Figure 5b). The location of the bottleneck of the p2 tunnel in AncLinB-DmbA is close to the entrance to the surface when compared to its descendants. The p2 tunnel of AncLinB-DmbA consists of 32 residues, with the bottleneck formed by D143, W144, P145, A248, I249, T251, M254, V250 with a radius of ∼1 Å. LinB has the same bottleneck radius. The bottleneck radius of the p2a and p2b tunnels in DmbA is the smallest, both are at ∼0.9 Å.

The p1 tunnel entrance is located between the α4-helix and the β7- and β8-sheets in all compared proteins. The p2 tunnel entrance is located near the α9 in all proteins, however, the p2b tunnel entrance in DmbA is located between α2 and α8 on the opposite side of the molecule (Figure 5g). The radius of the entrance in AncLinB-DmbA is the largest among the descendants: it is ∼2.5 Å, compared to DmbA ∼2.2 Å and LinB ∼1.4 Å (Table 2). The entrance to the p1 tunnel additionally consists of residues P145, E147, A178, A272, G177, G247 (Figure 5a). The main difference in the p1 entrance among the tested enzymes is represented by the negatively charged residue E147 in AncLinB-DmbA, while to nonpolar Q146 in LinB and the nonpolar hydrophobic A145 in DmbA. Another major difference is the location of L177 in LinB (Figure 5b), while both DmbA and AncLinB-DmbA contain A178 (Figure 5a,c). The surface residue L177 in LinB is located in the mouth of the p1 tunnel where its side chain partially blocks the entrance of the substrates inside the enzyme active site. The presence of bottleneck residue L177 thus makes the bottleneck radius of LinB p1 tunnel the smallest when compared with AncLinB-DmbA and DmbA. The sidechain of D147 in LinB faces tunnel p1 and thus forms another bottleneck residue together with L177. It can be concluded that these two residues are responsible for the narrowest bottleneck in LinB, compared to other tested enzymes. Both D147 and L177 are important determinants of substrate specificity of LinB and therefore have been subjected to mutagenesis [35]. Other amino acids involved in the p1 tunnel formation are similar to each other and probably do not play a crucial role in enzyme functionality. The difference in entrance composition of p2 tunnel in AncLinB-DmbA is represented by bottleneck residue M254, where this residue is replaced by R253 and R252 in DmbA and LinB respectively. The other bottleneck residues of p2 tunnel in AncLinB-DmbA are L139 and with I249. In DmbA, the bulky M254 is replaced by I254 and thus the bottleneck is formed only by M139 and I249 resides. In LinB, M253 forms a bottleneck of the p2 tunnel together with I138 and L248 (Figure 5d,e). Another difference is W144 residue present in AncLinB-DmbA and DmbA in both tunnels p1 and p2a and F143 in LinB. At this position, the curvature of p2a in AncLinB-DmbA and DmbA is higher than in LinB (Figure 5d–f), which might indicate that W144 is responsible for the flexure. The other residues, participating in the p2a tunnel formation are the same or similar in all compared proteins.

The comparison of tunnels length revealed the longest p1 tunnel in LinB ∼6.9 Å. The shorter tunnels are in AncLinB-DmbA and in DmbA, both ∼3.9 Å. The length of p2 tunnels showed different situations: DmbA slot tunnels have length p2a ∼19.9 Å and p2b ∼24.3 Å; LinB p2a ∼17 Å (Table 2). The longest p2a tunnel length is in AncLinB-DmbA ∼22.4 Å. Due to a big curvature of AncLinB-DmbA, its p2a slot tunnel has the shortest distance to the surface among the others (Table 2). The overall tunnel properties of AncLinB-DmbA correlate more with DmbA than with LinB. The chemical properties of AncLinB-DmbA residues composing entrance to the p1 and p2a tunnels probably play a vital role in the substrate specificity of this protein.

The active site cavity is located between two domains in all dehalogenases and its volume is important in substrate specificity [36]. The volume of the active site cavity of AncLinB-DmbA is 460 Å^3^, which is larger compared to both LinB (406 Å^3^) and DmbA (375 Å^3^) (Table 2). Previous studies have demonstrated a correlation of active site cavity volume and access radius with substrate specificity in haloalkane dehalogenases [37,38]. The fact that AncLinB-DmbA has the biggest cavity volume leads to the conclusion that this enzyme exhibits a broader substrate specificity.

Finally, the structure of AncLinB-DmbA was solved and it shares a high degree of similarity with closely related modern-day haloalkane dehalogenases. Despite the highly conserved structure of AncLinB-DmbA and structural similarities with LinB and DmbA, the ancestral enzyme exhibits some differences in the overall structure. This is embodied in variations of α-helices and β-sheets sizes and positions as well as the positioning of more flexible elements such as loops. Caver analysis revealed differences in several residues responsible for tunnels formation. Nevertheless, the core residues correspond to those in both LinB and DmbA. The differences in access tunnels of compared proteins revealed some advantages of AncLinB-DmbA from its descendants. The bottleneck radius in the p1 tunnel of AncLinB-DmbA is close to the wider bottleneck in DmbA. However, the ancestral protein revealed the widest entrance radius of the main tunnel among the other two proteins. The p2a bottleneck radius of AncLinB-DmbA has the same radius as LinB and from all compared proteins AncLinB-DmbA p2a bottleneck is located at the entrance of the tunnel. The biggest active site cavity was revealed in the AncLinB-DmbA. The largest active site cavity was found in AncLinB-DmbA. The knowledge of the composition of tunnel residues in AncLinB-DmbA may represent an advantage in further modification of its catalytic properties.

## 3. Materials and Methods

### 3.1. Ancestral Sequence Reconstruction and Gene Synthesis

The ancestral sequence was reconstructed as previously described [39,40,41]. Protein sequences for the HLD-II subfamily were identified by database searching followed by clustering. The final nonredundant HLD-II dataset comprised several sequences and was used to infer the maximum likelihood phylogenetic tree of the HLD-II subfamily. The topology of the HLD-II tree agreed with the relevant parts of a previously published HLD family tree. The ancestral nodes along the evolutionary lineage to LinB and DmbA were predicted and the most common ancestor of the two enzymes was selected for laboratory resurrection. The selected node, the most probable ancestor sequence, was predicted by assigning to each position the ancestral state with the highest-weighted posterior probability. Positions with posterior probability less than 90% in the most likely ancestral state were considered ambiguous. Gene encoding inferred sequence of AncLinB-DmbA was synthetized artificially (GeneArt, Life technologies, Regensburg, Germany). The codon usage was automatically adapted to the codon bias of *Escherichia coli* genes by GeneArt’s website service. For expression purposes, the AncLinB-DmbA gene was subcloned into the expression vector pET21b (Novagen, San Diego, CA, USA) between NdeI and BamHI restriction sites (Table 3).

### 3.2. Protein Expression and Purification

To overproduce AncLinB-DmbA in *E. coli*, expression of the corresponding gene (under control of the T7lac promoter) was induced by adding isopropyl β-D-thiogalactopyranoside (IPTG). *E. coli* strain BL21(DE3) cells containing recombinant plasmid pET21b: *ancLinB-DmbA* were grown in Luria broth medium containing ampicillin (100 µg/mL) at 37 °C. When the cell culture reached an optical density of 0.6 at a wavelength of 600 nm, gene expression was induced by the addition of IPTG (final concentration 0.5 mM) and the cells were cultivated overnight at 20 °C. The cells were harvested, disrupted by sonication using a UP200S ultrasonic processor (Hielscher, Teltow, Germany), and centrifuged for 1 h at 4 °C and 21,000× *g*. The supernatant was collected and further purified on a HiTrap IMAC HP 5 mL column charged with Ni^2+^ ions (GE Healthcare, Uppsala, Sweden). The His-tagged enzyme was bound to the resin in an equilibrating buffer (20 mM potassium phosphate buffer, pH 7.5, containing 0.5 M sodium chloride and 10 mM imidazole). Unbound and nonspecifically bound proteins were eliminated by washing with a buffer containing 50 mM imidazole. The enzyme was eluted by a buffer containing 300 mM imidazole. The active fractions were pooled and dialyzed against 50 mM potassium phosphate buffer (pH 7.5) at 4 °C. The Bradford reagent (Sigma-Aldrich, St. Louis, MO, USA) was used to determine the enzyme concentration, with bovine serum albumin used as a standard. Enzyme purity was checked by sodium dodecyl polyacrylamide gel electrophoresis.

### 3.3. Crystallization

The crystallization process was performed manually in CombiClover crystallization plates (Emerald Biosystems, Bainbridge Island, WA, USA) by the sitting drop vapor diffusion method [42]. PegRx and Crystal screen (Hampton Research, Aliso Viejo, CA, USA) as well as NeXtal DWBlock Suites (Quigen, Crawley, UK) commercial crystallization screens were used. Finally, the crystals grew in the crystallization screen Crystal screen (Hampton Research, Aliso Viejo, CA, USA). One microliter of protein solution at a concentration of 9.8 mg/mL in a 50 mM Tris–HCl buffer pH 7.5 was mixed with a reservoir solution in the ratios 1:1 and 1:2 and equilibrated against 500–1000 μL of the reservoir solution. The crystals grew with a size appropriate for synchrotron data collection without any further optimization.

### 3.4. Data Collection

The diffraction dataset was collected at the BESSY-II electron storage ring (Berlin-Adlershof, Germany) by the macromolecular crystallographic beamline 14.1, operated by the Helmholz-Zentrum Berlin [43], equipped with a PILATUS detector (Dectris, Baden, Switzerland).

The crystals of AncLinB-DmbA were mounted in Litholoops (Molecular Dimensions Limited, Sheffield, UK) or nylon cryoloops (Hampton Research, Aliso Viejo, CA, USA) and then flash-cooled in liquid nitrogen. Diffraction experiments were performed at 100 K. All the data collection statistics are summarized in (Table 1).

### 3.5. Structure Solution and Refinement

The dataset was indexed and integrated using the XDS software package [44], and scaled using the program Scala from the CCP4 program package [28]. The structure was solved using the molecular replacement method by the MOLREP program [45]. The structure was refined by the REFMAC5 program [46] and manual building in COOT [29]. Figures with structural representations were prepared using PyMOL [47]. The data refinement statistics are summarized in (Table 1). The Caver Web v.1.0 program was used for tunnel detection and visualization [34].

## 4. Conclusions

In summary, the structural analysis of the reconstructed ancestral enzyme AncLinB-DmbA was performed, and the crystal structure was compared with closely related descendants LinB and DmbA. Despite high sequence similarities, all three proteins exhibited structural differences in the size and special arrangement of some secondary structure elements in both the main and the cap domains. Significant differences in the architecture of the access tunnels were also found. The tunnels of AncLinB-DmbA are more similar to those of DmbA in size and number of residues. The physical properties (length, curvature, radius of bottlenecks, and active site cavity volume) of tunnels in AncLinB-DmbA show that this protein has the best characteristics from its descendants. AncLinB-DmbA was found to have the largest volume of the active site cavity and the largest entrance radius of the p1 main tunnel among the compared proteins. In addition, the curvature of the slot p2a channel in AncLinB-DmbA is the highest in comparison to LinB and DmbA. In conclusion, the structural information on AncLinB-DmbA reported here improves the understanding of the enzyme properties and supports the method of ancestral sequence reconstruction as a valuable tool for enzyme modification.

## Figures and Tables

**Table 1:** The data collection and refinement statistics for AncLinB-DmbA.

Data Collection	
Space Group	*P*4_3_22
*a*, *b*, *c* (Å)	68.89, 68.89, 156.24
α, β, γ (°)	90.0, 90.0, 90.0
Resolution range (Å)	50–1.50 (1.59–1.50)
Total no. of reflections	641,156 (102,219)
No. of unique reflections	61,367 (9534)
Completeness (%)	99.6 (97.8)
〈*I*/σ(*I*)〉	13.68 (1.65)
*R_meas_* *	102.9 (9.6)
CC_1/2_	0.99 (0.77)
Overall *B* factor from Wilson plot (Å^2^)	27.062
Refinement	
No. of reflections used for refinement	58,297
*R_work_* ^‡^/*R_free_* ^§^ (%)	15.94/17.10
No. of non-H atoms	2810
No. of protein atoms	2475
No. of chloride ions	5
No of ligands	1
No. of water molecules	319
Average B factor (Å^2^)	22.357
Ramachandran plot	
Most favored (%)	95.89
Allowed (%)	3.79
Outliers (%)	0
R.m.s. deviations	
Bonds (Å)	0.019
Angles (°)	1.958
PDB ID	7PW1

* *R_meas_* is a redundancy- independent merging R factor, $R_{meas}=\underset{hkl}{\sum }{\left\{N\left(hkl\right)/\left[N\left(hkl\right)-1\right]\right\}}^{1/2}\underset{i}{\sum }\left|I_{i}\left(hkl\right)-\langle I(hkl)\rangle \right|/\underset{hkl}{\sum }\underset{i}{\sum }I_{i}\left(hkl\right)$, where 〈I(hkl)〉 is the mean of the N(hkl) individual measurements $I_{i}\left(hkl\right)$ of the density of reflections  hkl., ^‡^ $R_{work}=\underset{hkl}{\sum }\left|\left|F_{obs}\right|-\left|F_{calc}\right|\right|/\underset{hkl}{\sum }\left|F_{obs}\right|$, ^§^ *R_free_* was calculated using 5% of the reflection data that were excluded from refinement.

**Table 2:** The properties and tunnel composition of selected haloalkane dehalogenases.

	AncLinB-DmbA	LinB	DmbA
PDB code	7PW1	1CV2	2QVB
Resolution (Å)	1.5	1.58	1.19
Sequence identity to AncLinB-DmbA (%)	-	81.7	83.2
R.m.s.d. for AncLinB-DmbA	-	0.778	0.995
Active site cavity volume (Å^3^)	460	406	375
p1 main tunnel characteristics			
Bottleneck radius (Å)	2.0	1.4	2.1
Number of residues	17	22	18
Length (Å)	3.9	6.9	3.9
Entrance radius (Å)	2.5	1.4	2.2
Distance to surface * (Å)	3.9	6.7	3.5
Curvature ^‡^	1	1	1.1
p2a slot tunnel characteristics			
Bottleneck radius (Å)	1	1	0.9
Number of residues	32	34	30
Length (Å)	22.4	17	19.9
Entrance radius (Å)	1.0	1.7	1.6
Distance to surface * (Å)	8.1	11.5	11.3
Curvature ^‡^	2.8	1.5	1.8
p2b slot tunnel characteristics			
Bottleneck radius (Å)	-	-	0.9
Number of residues	-	-	36
Length (Å)	-	-	24.3
Entrance radius (Å)	-	-	1.6
Distance to surface * (Å)	-	-	19.4
Curvature ^‡^	-	-	1.3

* Distance to surface is a shortest length from the starting point (active site) and the surface in the direction of the tunnel. ^‡^ Curvature as a description of the shape of the tunnel as the ratio between the length of the tunnel and the shortest possible distance between the starting point and the tunnel ending point.

**Table 3:** Production specifics for AncLinB-DmbA.

Source Organism	Artificial Gene
DNA source	-
Restriction sites	*Nde*I/*BamH*I
Vector	pET21b
Expression host	*E. coli*
Complete amino acid sequence of the construct produced	MTALGAEPYGQKKFIEIAGKRMAYIDEGEGDPIVFQHGNPTSSYLWRNIMPHLEGLGRLIACDLIGMGDSDKLSPSGPDRYSYAEHRDYLFALWEALDLGDNVVLVIHDWGSALGFDWANQHRDRVQGIAYMEAIVTPLEWADWPEEVRDIFQGFRSPAGEEMVLENNIFVERVLPGAILRQLSDEEMAEYRRPFLNAGEDRRPTLSWPRQIPIDGEPADVVAIVSDYASWLAESDIPKLFINAEPGAIVTGRMRDFCRSWPNQTEITVKGAHFIQEDSPDEIGAAIAEFVRRLRAAAGV

## Data Availability

The data presented in this study are available on request from the Corresponding author.
